# How to use luspatercept and erythropoiesis‐stimulating agents in low‐risk myelodysplastic syndrome

**DOI:** 10.1111/bjh.20126

**Published:** 2025-05-02

**Authors:** Valeria Santini, Angela Consagra

**Affiliations:** ^1^ MDS Unit, DMSC‐Hematology University of Florence, AOU Careggi Florence Italy

**Keywords:** anaemia, erythropoietin, ESAs, low‐risk myelodysplastic syndromes, luspatercept, transfusion dependence

## Abstract

Anaemia is the most common cytopenia in myelodysplastic syndrome (MDS), significantly impacting quality of life and morbidity. Erythropoiesis‐stimulating agents (ESAs) are the first‐line treatment for anaemia in lower risk (LR)‐MDS. The European Medicines Agency (EMA) approved epoetin alpha for LR‐MDS‐related anaemia in 2017, based on evidence from a unique randomized Phase 3 trial and accumulated evidence in many trials, providing support to an already widely utilized therapeutic option. Luspatercept, a more recently approved agent, is a ligand trap for transforming growth factor beta (TGF‐β) pathway, whose activation is associated with impaired terminal erythroid maturation in MDS. Luspatercept facilitates late‐stage erythroid differentiation, improving transfusion‐dependent anaemia in LR‐MDS, and has shown activity after ESA failure in MDS‐ring sideroblasts (RS) and in all subtypes of MDS ESA naïve transfusion‐dependent patients. Due to the recent approval of luspatercept also in ESA naïve LR‐MDS, it has become crucial to determine the optimal treatment algorithm for anaemic LR‐MDS, before and after transfusion dependence. ESAs and luspatercept are characterized by distinct mechanisms of action, and their integration into treatment strategies is already possible, but requires further evidence to maximize efficacy and improve patient outcomes.

## INTRODUCTION

Myelodysplastic syndromes/neoplasias (MDS) are a constellation of different diseases presenting anaemia as the most frequent cytopenia.^1^ In fact, anaemia shapes the clinical presentation and severity of the disease. Different classifications as well as prognostic systems have been proposed over the years, along with the increased knowledge on the nature and clinical behaviour of MDS.^2^, ^3^, ^4^, ^5^, ^6^, ^7^, ^8^, ^9^ The majority of MDS cases belong to the prognostic category defined ‘lower risk’ (LR) according to Revised‐International Prognostic Scoring System (IPSS‐R).^7^ These patients have a reasonably long life expectancy, but very often become red blood cell (RBC) transfusion dependent (TD).

Anaemia in MDS is due to ineffective erythropoiesis, with apoptosis of marrow erythroid progenitors. The pathophysiology of anaemia of MDS is complex: First of all, there is a high degree of heterogeneity in the prevalent erythroid subpopulation affected by the neoplastic alterations, indicating that the dysplastic erythroid progenitor is not the same for different subtypes of MDS. It has to be kept in mind that many concomitant causes can aggravate anaemia and may further contribute to the deterioration of erythropoiesis. Reducing anaemia is thus a key treatment goal in LR‐MDS.^10^, ^11^, ^12^ In the Italian MDS Registry FISiM (Fondazione Italiana Sindromi Mielodisplastiche), as much as 63% of patients have concomitant diseases (www.fisimematologia.it). In anaemic elderly patients, comorbidities may account for the severity of symptoms. Because of that, the clinical priority in anaemic MDS patients is the improvement of haemoglobin (Hb) levels.

For more than two decades, symptomatic anaemia in LR‐MDS has been treated with erythropoietic‐stimulating agents (ESAs). Paradoxically, erythropoietin (EPO) has been officially approved only a few years ago, after a tardive registration trial.^13^ Such a trial was designed with evident biases, agnostic of the evidence accumulated during the previous years of clinical application of ESAs. The use of ESAs is at present a common routine. Real‐world practice has confirmed the efficacy of these agents and unfortunately also the dismal prognosis of patients who lose response and become transfusion dependent. In fact, even in first‐line therapy, not all LR‐MDS patients respond to ESAs treatment and, even so, response is not permanently maintained. Given the availability of luspatercept, treating physicians require a critical evaluation supported by clear evidence to guide the choice between these two therapeutic options in what might appear to be a homogeneous population of MDS patients.

## ESAs AND BIOSIMILARS

Erythropoiesis‐stimulating agents, including recombinant human EPO (epoetin alfa, beta, zeta and theta) and the longer acting darbepoetin alfa, are the cornerstone of anaemia treatment in LR‐MDS (Table 1), seemingly promoting the proliferation of erythroid cells and preventing the intramedullary apoptosis of erythroid precursors, thereby addressing ineffective erythropoiesis. The activity of ESAs and biosimilars in anaemic MDS patients is confirmed by data obtained from a couple of randomized clinical trials, observational studies, both prospective and retrospective, and reinforced by well‐conducted meta‐analyses. These studies have explored the therapeutic potential of ESAs, administered either alone or, although more rarely, in combination with growth factors like granulocyte colony‐stimulating factor (G‐CSF).^14^, ^15^, ^16^, ^17^, ^18^, ^19^, ^20^

**TABLE 1:** Erythropoietic stimulating agent therapy in LR‐MDS.

ESA type	Dosing schedule	Efficacy (response rate), %	Limitations	Comparison and combination studies
Epoetin alfa	30 000–40 000 IU SC weekly	~40–60	Lower response in patients with high endogenous EPO (>500 U/L)	The EPOANE3021 trial showed significant efficacy over placebo (31.8% vs. 4.4%)
Epoetin beta	30 000–60 000 IU SC weekly	~40–60	Requires frequent administration	A randomized study showed that adding G‐CSF improved response rates (33.3% vs. 62.5%, *p* = 0.03)
Darbepoetin alfa	150–300 μg SC every 2–3 weeks	~50–70	More expensive than epoetin; response duration varies	The ARCADE trial showed superior transfusion reduction over placebo (36.1% vs. 59.2%)
Methoxy polyethylene glycol‐epoetin beta (CERA)	360 μg SC every 3–4 weeks	~40–60	Less commonly used in MDS; limited comparative data	Not widely studied in MDS, but long‐acting profile offers potential benefits
Erythropoietin‐zeta	40 000–80 000 IU SC weekly	~50–55	Less commonly used in MDS; limited comparative data	Not widely studied in MDS, but is a valid and safe alternative

*Note*: This table summarizes the dosing schedules, efficacy and limitations of different ESAs used in LR‐MDS. It also highlights key findings from comparative and combination studies. ESA type: different formulations of erythropoietin used in clinical practice. Dosing schedule: standard SC administration regimens. Efficacy (response rate): approximate percentage of patients showing haematological improvement or transfusion independence. Limitations: factors that may affect ESA response, such as high endogenous EPO levels, cost or need for frequent administration. Comparison and combination studies: major clinical trials evaluating ESA efficacy, either alone or in combination with other agents like G‐CSF.

Abbreviations: EPO, erythropoietin; ESAs, erythropoiesis‐stimulating agents; G‐CSF, granulocyte colony‐stimulating factor; LR‐MDS, low‐risk myelodysplastic syndromes; MDS, myelodysplastic syndromes; SC, subcutaneous.

Two mechanisms of action of ESAs are supposedly possible, but evidence is lacking on whether the target cells are selectively the dysplastic or the residual normal ones, or even both.^21^ First of all, ESAs may reduce apoptosis and support the survival of erythroid progenitors. ESA stimulation can facilitate proliferation, differentiation and the restoration of erythropoiesis within the MDS clone.^21^ We observed several years ago that CD71‐positive bone marrow erythroid progenitors from MDS patients fail to activate signal transducer and activator of transcription 5 (STAT5) after in vitro EPO stimulation, and this lack of activation correlates with clinical unresponsiveness to ESAs.^22^

In fact, ESA's activity in MDS is impaired by the presence of a defective EPO signalling pathway, which reflects the intrinsic abnormalities of the erythroid progenitors. Despite expressing normal levels of EPO receptors,^23^ dysplastic erythroid progenitor cells in MDS exhibit defective downstream signalling.^24^ A key defect involves the lack of STAT5 phosphorylation, a critical step for erythroid maturation.^24^ This defect was initially attributed to structural abnormalities in EPO receptors, but the hypothesis remains unproven.^25^ Other studies have identified defects in extracellular signal‐ regulated kinase (ERK1/2) phosphorylation upon ESA in vitro exposure, emphasizing the broader disruption of intracellular signalling.^26^ Additionally, MDS progenitors demonstrate spontaneous p38 mitogen‐activated protein kinase (MAPK) activation, further indicating aberrant signalling pathways.^27^ Previous findings suggested that impaired formation of lipid raft microdomains in MDS erythroid progenitors contributes to defective EPO signalling.^28^ These lipid rafts, which assemble key signalling components such as the EPO receptor, STAT5, Janus Kinase 2 (JAK2) and Lck and Yes novel tyrosin kinase (Lyn) kinase, are essential for effective EPO receptor function. In MDS, erythroid progenitors exhibit significantly reduced lipid raft assembly and smaller raft aggregates, compromising EPO signalling and potentially limiting the efficacy of ESAs.^28^, ^29^

The second mechanism suggested implies that ESAs could stimulate polyclonal erythropoiesis by promoting the survival of residual normal erythroid precursors.^21^ The observation that early treatment with ESAs may achieve higher efficacy, because supposedly normal erythroid precursors are still present, may support this hypothesis, although clear evidence has not been produced yet.

Recommendations for the use of ESAs in managing anaemia in MDS patients have been issued by several organizations, including the Italian Society of Hematology (SIE), the British Society of Hematology, the National Comprehensive Cancer Network (NCCN) and the European Society for Medical Oncology (ESMO). The last ESMO guidelines for anaemia,^30^ although defined several years ago, encompass also specific recommendations for MDS patients. According to these guidelines and to the European approval, ESA therapy is suggested for MDS patients who meet the following criteria: symptomatic anaemia, Hb levels <10 g/dL, low to intermediate‐1 IPSS^6^ or very low‐ to intermediate‐risk IPSS‐R,^7^ <2 RBC units per month and/or serum EPO (sEPO) levels <500 iu/L (noting that the European Medicines Agency [EMA]‐approved indication for epoetin alpha specifies the need for sEPO level of <200 iu/L).

While most clinical trials investigating ESAs in lower risk MDS patients have focused on epoetin alfa, the efficacy of other ESAs in this context has also been established. Several studies have reported clinically significant responses with the use of epoetin beta,^31^, ^32^ darbepoetin alpha^33^, ^34^, ^35^, ^36^ and epoetin zeta^37^ (Table 4). It was only in March 2017 that the EMA approved the use of epoetin alfa to treat anaemia in LR‐MDS, based on the only Phase 3 randomized clinical trial.^13^ The aforementioned study suffered from several problems: patients presenting de novo IPSS^6^ low or intermediate‐1 MDS, with Hb ≤10.0 g/dL could be enrolled even if they were transfusion dependent. This inclusion was possible despite the fact that transfusion dependence is a known variable correlated with scarce response to ESAs. Patients eligible for this study had a transfusion burden of ≤4 RBC units within 8 weeks before randomization, and they could present with sEPO <500 mU/mL. Moreover, treatment was frequently interrupted when Hb >11 g/dL, so that the criteria of response could not always be met. The patients had to be treated with a weight adjusted and not fixed dose of epoetin alfa (450 iu/kg, weekly subcutaneous) and compared with placebo. Notwithstanding that, the primary end‐point^38^ was met: erythroid response at week 24 (per International Working Group [IWG] 2006 criteria^38^) was 31.8% in the epoetin alfa group vs. 4.4% in the placebo group; *p* < 0.001.

Although different and not standardized criteria and end‐points were applied to the numerous studies performed, a pooled analysis of 1587 patients with LR‐MDS demonstrated ESA response rate of 39.5%, based on Hb increases of 1 g/dL or greater or achievement of transfusion independence (TI).^39^ A randomized, blinded trial of darbepoetin alfa in 147 LR‐MDS patients with Hb <10 g/dL and sEPO <500 iu/L reported a haematological improvement of 14.7%, compared to 0% in the placebo group (*p* = 0.02).^36^

Higher doses of ESAs are typically required in MDS, compared to doses used for anaemia in chronic kidney disease. Recombinant human EPO is commonly administered at 30 000/80 000 units weekly, while darbepoetin alfa is given at 500 μg every 3 weeks.^34^, ^36^ The relevance of higher doses than 30–40 000 U/week is debatable.^40^ In an early piece of work, EPO 40 000 U biweekly as induction therapy followed by 40 000 U weekly maintenance was demonstrated effective in rapidly correcting anaemia, improving quality of life (QoL) and reducing transfusion requirements in LR‐MDS patients with Hb <10 g/dL.

We observed no differences between different ESA doses (*p* = 0.28) in paired matched cohorts, especially when evaluating effects on overall survival (OS).^41^ It is important to initiate ESA therapy early, ideally before the need for transfusion arises. In general, response to ESAs is seen within 12 weeks of treatment and lasts a median of 17–24 months.^42^, ^43^ To maintain this response, clinicians should monitor and correct deficiencies in iron, vitamin B12 and folate levels. Discontinuation of ESA therapy invariably leads to the loss of response.^43^

Erythropoiesis‐stimulating agents are generally well‐tolerated, and adverse events such as thrombotic events and hypertensive episodes are rare, provided Hb levels are kept <12 g/dL. In particular, thrombotic events in MDS patients have been evaluated in a study based on Medicare data which have shown that only a minority of cases of MDS receiving ESA reported thrombotic events, all of them having a predisposing condition like insertion of central venous catheter or frequent RBC transfusions.^44^ These observations suggest particular caution in the use of ESAs in patients with a previous history of thrombosis.

Despite their efficacy, ESAs exhibit a transient activity, with a duration of response of 6–24 months, and many patients eventually will require chronic transfusion support.^42^

The achievement of erythroid improvement (HI‐E) and maintenance of TI by ESA treatment has an impact on OS. In fact, it is well known that TI correlates with longer OS, and our group demonstrated in 1145 cases of LR‐MDS that a favourable effect of ESA (compared to non‐treatment) was suggested for patients with Hb 8–10 g/dL. This effect was present for patients with a diagnosis of refractory anaemia (RA), refractory anaemia with ring sideroblasts (RARS) according to the WHO 2008 classification (that at present would be MDS‐low blasts (LB) and MDS‐ring sideroblasts [RS], respectively, according to the WHO 2022) or MDS with isolated deletion of chromosome 5q (MDS‐del(5q)).^45^, ^46^ In specific, there are scarce data on comparative OS. Randomized studies of ESAs versus supportive care are very rare, and most of them have follow‐up that are too short to evaluate OS in LR‐MDS. When patients receiving ESAs have been considered irrespective of response, median survival has been found only numerically higher than that observed for supportive care.^18^ On the other hand, achievement of HI‐E resulted in superior OS.^41^, ^45^

## PREDICTIVE FACTORS OF RESPONSE TO ESAs

Response to ESAs in LR‐MDS is influenced by a combination of patient‐specific factors, including disease characteristics, transfusion history and specific haematological markers (Table 2). Over the years, the variables associated with response of resistance have been clarified, although not completely. Poor or absent response to ESAs is correlated with transfusion dependence, defined as receiving chronically >2 RBC units in 8 weeks, high serum levels of endogenous EPO (>200 U/L and more so >500 U/L), bone marrow blasts >5%, complex karyotype, high ferritin levels, multilineage dysplasia and higher risk classifications according to the IPSS‐R.^7^, ^47^, ^48^ If patients are selected for ESA treatment taking into account all the above‐mentioned variables, especially very low IPSS‐R risk score, the percentage of response can reach 85%.^48^, ^49^

**TABLE 2:** Predictive factors of response to ESAs in LR‐MDS.

Factor	*p*‐Value	Reference
Clinical parameters		
Serum EPO <200 U/L	<0.001	19, 33
Low transfusion burden (<2 RBC units/8 weeks)	<0.001	19, 47
Normal or low ferritin levels	N/A	19, 48
Morphological and MFC parameters		
Bone marrow blasts <5%	0.0052	48
Absence of multilineage dysplasia	N/A	45
CD117^+^ erythroid precursor	0.019	50
Genetic and molecular parameters		
Low mutational burden (<2 mutated genes)	0.01	51
Absence of complex karyotype	0.008	48
IPSS‐R very low/low risk	<0.001	48
IPSS‐M very low/low risk	<0.0001	52

*Note*: This table summarizes the main predictive factors of response to ESAs in patients with low‐risk myelodysplastic syndromes (LR‐MDS). The factors are categorized into clinical, morphological, immunophenotypic, genetic and molecular parameters. The absence of these predictive factors of positive response is generally associated with non‐response or a shorter response duration. The *p*‐values reported refer to univariate analyses; for details on multivariate analyses or further insights, please refer to the corresponding references.

Abbreviations: EPO, erythropoietin; IPSS‐M, Molecular International Prognostic Scoring System; IPSS‐R, Revised International Prognostic Scoring System; MFC, multiparametric flow cytometry; N/A, not applicable; RBC, red blood cells.

We recently showed that the best ESA responders are LR‐MDS patients with lower risk disease according to the Revised International Prognostic Scoring System for Molecular Analysis (IPSS‐M).^9^, ^52^ It comes as a consequence that treatment with ESAs should be implemented as soon as anaemia becomes symptomatic and <10 g/dL and before additional somatic mutations occur and clonal progression takes place.^19^, ^48^ There are at present no evidences of specific mutations associated with response or lack of response to ESAs, but it is their number and the molecular risk score that correlate with it. This suggests that IPSS‐M scoring may be useful also in the setting of evaluation of treatment with ESAs.

As mentioned above, elevated levels of sEPO (>200 U/L) are typically associated with lack of response, reflecting a compensatory mechanism for severe anaemia that points to a rather long history of the disease.^19^, ^33^ Although not applicable in routine practice, it has been observed that pro‐inflammatory cytokines, and inflammation in general, are associated with lower ESA responsiveness.^53^, ^54^ More recent evidence in this sense is lacking, although anti‐inflammatory approaches are ongoing for treatment of anaemic LR‐MDS. Advanced age is not influencing response to ESAs.^55^

All the variables above mentioned as predictive of ESAs response/refractoriness are consistent with the concept that a less advanced disease with possible residual normal haematopoiesis is more prone to respond.^47^ The relevance of intrinsic defects in intracellular signalling typical of dysplastic erythroid progenitors is supportive of this concept.^26^

Both iron deficiency and iron overload can impair ESAs response. It is thus mandatory to evaluate and correct functional iron deficiency before inception and during ESA treatment.^56^

Response to ESAs in LR‐MDS is influenced by the interplay of mutational profiles and erythroid precursor characteristics. As alluded to above, recent data from our group^52^ emphasize the importance of mutational burden^51^ and more so of the IPSS‐M^9^ score in predicting ESAs' responsiveness. Lower risk scores IPSS‐M and especially the absence of high‐risk mutations indicated by IPSS‐M are associated with a favourable response to ESAs, underscoring the value of genetic profiling even when planning ESA treatment, to avoid useless and long treatments.

The Spanish MDS group (GESMD) recently observed a trend to a higher frequency of erythroid response among patients with a lower number of mutated genes (40.4% in <3 mutated genes vs. 22.2% in ≥3; *p* = 0.170). LR‐MDS patients with <3 mutations had also longer OS, consistent to what we demonstrated.^51^

In association with molecular evaluation, MDS responders to ESAs have a higher proportion of immature erythroid precursors (CD117+).^50^ Therefore, the cytofluorimetric profiling of the marrow erythroid compartment may support the choice of therapy. In conclusion, IPSS‐M stratification and cytofluorimetric analysis may improve the predictive assessment of ESA susceptibility at baseline (Table 2).

## LUSPATERCEPT

The transforming growth factor beta (TGF‐β) superfamily signalling pathway plays a vital role in regulating haematopoiesis and in specific erythropoiesis. This pathway involves receptor ligands such as activins and growth differentiation factors (GDFs), which influence cellular processes including apoptosis, proliferation, differentiation and migration.^57^ Under normal physiological conditions, TGF‐β signalling functions as a myelosuppressive factor by inhibiting erythroid differentiation via apoptosis and cell cycle arrest in erythroblasts.^57^ Consequently, erythroid maturation depends on the simultaneous suppression of TGF‐β signalling and stimulation by EPO.

The ligands of the TGF‐β receptor family are polypeptide growth factors, including TGF‐β, activins, bone morphogenetic proteins (BMPs) and growth differentiation factor 11 (GDF11).^58^ Within this pathway, small mother against decapentaplegic (SMAD) proteins act as key regulators of haematopoiesis. Upon ligand binding and receptor activation, signalling is initiated, with SMAD2/3 and SMAD1/5/8 mediating canonical intracellular signalling through ligand–receptor complexes.^58^

In MDS, SMAD2/3 downstream mediators are persistently activated and overexpressed in erythroid cells, contributing to ineffective erythropoiesis. Interestingly, studies have demonstrated that pharmacological inhibition of TGF‐β receptors or suppression of SMAD2/3 activity using short hairpin RNA can enhance haematopoiesis across various MDS subtypes in vitro. This highlights the therapeutic potential of targeting the TGF‐β signalling pathway in managing MDS‐related dyserythropoiesis.^59^

Luspatercept is a fusion protein composed of a modified extracellular domain of the human activin receptor type IIB linked to the Fc domain of human IgG1, which serves as a stabilizer.^60^ Luspatercept has been first evaluated in MDS in a Phase 2 study, the PACE‐MDS trial^61^ after the serendipitous observation of an increase in Hb in women treated with TGF ligand traps.^62^ Fifty‐eight LR‐MDS patients received subcutaneous doses of luspatercept every 3 weeks, with dose escalation. Among patients receiving higher doses (0.75–1.75 mg/kg), 63% achieved erythroid response, marked by reduced RBC transfusion dependence or increased Hb levels. Furthermore, patients with certain genetic mutations, such as *SF3B1*, or the presence of RS showed higher response rates. Building on these results, the Phase 3 MEDALIST trial^60^ enrolled 229 TD patients with LR‐MDS with RS (MDS‐RS). Patients were randomized 2:1 to receive luspatercept or placebo. The trial demonstrated the superiority of luspatercept in achieving TI for >8 weeks (37.9% vs. 13.2%) and 12 weeks (28.1% vs. 7.9%). Among patients treated with luspatercept, 52.9% achieved HI‐E during the first 24 weeks compared to 11.8% in the placebo group.

Because of the excellent results in MDS‐RS who had received prior ESA and lost response (97% of cases included in the study), a further phase 3 study investigating the activity of luspatercept versus epoetin alfa in erythropoiesis‐stimulating agent‐naive, TD, LR‐MDS was designed.^63^ An interim analysis in 2023 showed that luspatercept led to TI in 59% of patients compared to 31% in the epoetin alfa group. Interestingly, among patients with higher TB (HTB ≥4 RBC units per 8 weeks), luspatercept showed promising efficacy, with 45% of patients responding versus 20% in the epoetin alfa group. The final analysis of the study^64^ confirmed the superior achievement of TI >12 weeks and an increase of 1.5 g/dL in Hb level in patients treated with luspatercept versus ESA: patients in the luspatercept group reached the primary end‐point (60% vs. 35%). These results were irrespective of sEPO levels, presence of RS, TB. To the latter point, for patients with <4 RBC units/8 weeks, TI was 69%, whereas for patients receiving ≥4 RBC units/8 weeks, it was 37%. Safety analyses indicated common grade 3–4 treatment‐emergent adverse events occurring among luspatercept recipients (*n* = 182): hypertension and other cytopenias related to the baseline disease.

Based on the results of the MEDALIST study,^60^ luspatercept was approved in 2020 by the Food and Drug Administration (FDA)^65^ and EMA^66^ for the treatment of LR‐MDS‐RS according to the 2016 WHO classification.^3^ It is thus indicated for patients who require RBC transfusions (≥2 units every 8 weeks) following failure, refractoriness or ineligibility for ESA treatment. After the results of the COMMANDS trial,^63^ luspatercept was approved by the FDA^67^ in 2023 and by the EMA^66^ in 2024 for the treatment of all TD LR‐MDS, regardless of the presence of RS or the *SF3B1* mutation.

Luspatercept is administered via subcutaneous injection every 3 weeks; the starting dose is 1 mg/kg, with adjustments to 1.33 mg/kg after 6 weeks if there is no reduction in RBC transfusion need, and up to a maximum of 1.75 mg/kg if required. Pharmacokinetics are not significantly influenced by mild to moderate hepatic or renal impairment, baseline EPO or globulin levels, TB or concurrent use of iron‐chelating agents.

Similar to what is observed for ESAs, achievement of TI by luspatercept correlates with a prolonged OS in MDS‐RS patients treated in the MEDALIST trial.^68^

## PREDICTIVE FACTORS OF RESPONSE TO LUSPATERCEPT

In the real‐world (RW) data from the Mayo Clinic cohort^69^ of MDS‐RS patients treated with luspatercept, specific baseline factors showed a potential link to the response to therapy. Higher sEPO levels, particularly greater than 80 IU/L, were associated with a higher likelihood of response to luspatercept. In particular, 33% of patients with sEPO levels above 80 IU/L responded to luspatercpt, compared to none of the patients with sEPO levels < 80IU/L. Furthermore, the analysis of lymphocyte count (ALC) at the time of treatment initiation identified ALC ≥1.8 × 10^9^/L as a significant predictor of response. Notably, 75% of patients with ALC ≥1.8 × 10^9^/L responded to luspatercept, compared to just 9.4% in those with lower ALC.

A study conducted by FISiM^70^ further explored the relationship between baseline characteristics and the probability of achieving a TI response. In this study, baseline TB was a key predictor, with a significant correlation found between higher TB and the likelihood of achieving a primary response to treatment. However, no significant correlations were observed with factors such as age, gender, IPSS‐R^7^ or time since diagnosis. Our group also investigated possible factors correlated with response to luspatercept in a large cohort of treated LR‐MDS patients in haematological centres adhering to FISiM and in Moffitt Cancer Center.^71^ When examining the mutational patterns, a low IPSS‐M^9^ score was associated with a better treatment response. In the LR‐MDS cohort analysed in this study, neither the presence of specific *SF3B1* hot spot mutations nor their variant allele frequency (VAF) showed a correlation with response. However, stratifying patients by co‐mutation subgroups, as defined by IPSS‐M,^9^ revealed a trend of improved response in the *SF3B1* ^α^ group. This suggests that co‐mutation patterns may impact treatment outcomes in addition to their prognostic relevance. We validated the efficacy of luspatercept in an RW setting, consistent with findings from prior controlled studies^60^, ^63^ and identified baseline RBC TB as the strongest predictor of response. Predictive factors of response to luspatercept are summarized in Table 3.

**TABLE 3:** Predictive factors of response to luspatercept in LR‐MDS.

Factor	*p*‐Value	Reference
Clinical parameters		
Serum EPO >80 U/L	0.01	69
ALC ≥1.8 × 10^9^/L	0.005	69
Transfusion burden <4 RBC units/8 weeks	<0.001	70
Genetic and molecular parameters		
IPSS‐M lower scores	0.031	71
SF3B1 mutation presence	N/A	60, 63
SF3B1^α^ and SF3B1^β^ subgroup	0.046	63, 71

*Note*: This table summarizes the main predictive factors of response to luspatercept in patients with low‐risk myelodysplastic syndromes (LR‐MDS). The factors are categorized into clinical, genetic and molecular parameters. The absence of these predictive factors of positive response is generally associated with non‐response or a shorter response duration. The *p*‐values reported refer to univariate analyses; for details on multivariate analyses or further insights, please refer to the corresponding references. IPSS‐M lower scores refer to very low, low and intermediate–low risks; N/A, not applicable.

Abbreviations: ALC, absolute lymphocyte count; EPO, erythropoietin; IPSS‐M, Molecular International Prognostic Scoring System; RBC, red blood cells.

A randomized Phase 3 clinical trial (registered at ClinicalTrials.gov, NCT05949684) is ongoing to evaluate the efficacy of luspatercept versus ESAs in non‐transfusion‐dependent (NTD) LR‐MDS patients. A Phase 2 clinical trial^72^ evaluated the efficacy of luspatercept in a very small number of NTD LR‐MDS patients. The authors observed IWG‐defined^38^ HI‐E with >1.5 g/dL Hb increase in 47.6% of patients treated with luspatercept for 24 weeks.

Investigational data are also available exploring the potential synergy between ESAs and luspatercept,^73^ given their different and possibly complementary modes of action. The combination of the two agents could induce erythroid responses in patients, particularly those with suboptimal responses to monotherapy. While the findings of these sporadic studies are interesting, the evidence is still preliminary, and further evaluations are needed. In fact, an ongoing Phase 1/2 study (registered at ClinicalTrials.gov, NCT0581735) conducted by the Groupe Francophone des Myélodysplasies^74^ aims at evaluating the combination of luspatercept with ESA in ESA‐resistant or ineligible LR‐MDS patients at different doses of both agents. Eligible patients must have IPSS^6^ low‐ or intermediate‐1‐risk MDS without RS or del(5q) and Hb levels <9 g/dL and defined ineligible for or having failed ESA therapy in the absence of disease progression. In total, the results of 24 patients have been presented^74^: 29% of patients achieved erythroid response, with higher response rates observed in the higher dose cohorts. The optimal dose combination in the ongoing randomized Phase 2 study is luspatercept 1.75 mg/kg/3 weeks plus epoetin alfa 60 000 iu weekly.

### Combinations of ESAs and luspatercept

In the attempt to improve response and prolong duration of response, ESAs and luspatercept were combined, and both were also used with other agents with variable success (Table 4).^14^, ^47^, ^74^, ^75^, ^76^, ^77^

**TABLE 4:** Combination therapies in low‐risk MDS.

Combination therapy	Study details and patient population	Efficacy (response rate)	Key findings and benefits	Limitations
Epoetin beta + G‐CSF	Balleari et al.^14^, *N* = 30 ESA‐naïve patients	62.5% vs. 33.3%	Adding G‐CSF improved erythroid response significantly, especially in low EPO patients	Small study; low epoetin beta dose (30 000 iu/week)
Epoetin alfa + G‐CSF	Greenberg et al.^18^, *N* = 110 ESA‐failure patients	47% response after G‐CSF addition	G‐CSF was effective in patients who failed ESA alone	Retrospective study, heterogeneous patient population
Darbepoetin alfa + G‐CSF	Gotlib et al.^78^, Phase 2, *N* = 24 ESA‐failure patients	47% major erythroid response	G‐CSF addition improved response in ESA non‐responders	Small sample size
Darbepoetin alfa + G‐CSF	Houston et al.^79^, retrospective, *N* = 208	25% response in patients refractory to darbepoetin as single agent	G‐CSF was added in 14% of patients who did not initially respond to darbepoetin; 25% achieved response	Retrospective data; limited follow‐up
Epo + luspatercept	Komrokji et al.^73^, *N* = 28 ESA‐failure patients	36% obtained HI	Combination was effective in patients who lost response to luspatercept monotherapy	Small sample size; no control group
Epo + luspatercept	Adès et al.^74^, Phase 1–2 (GFM Combola Study)	Ongoing trial (NCT0581735)	Preliminary results suggest benefit in ESA‐failure non‐RS MDS patients	Ongoing study; final data pending
Epo + lenalidomide	Komrokji et al.^77^, lower‐risk MDS patients	Improved response in ESA‐refractory patients	Synergistic effect in MDS‐del(5q)	Needs further validation in non‐del(5q) patients
Epo + lenalidomide	Toma et al.^75^, ESA‐refractory MDS without 5q deletion	Improved response compared to ESA alone	Beneficial even in non‐del(5q) MDS	Study design limitations
Epo + lenalidomide	Van de Loosdrecht et al.^76^ (HOVON89 trial)	Significant increase in response for MDS‐del(5q)	Combination improved efficacy in MDS‐del(5q)	Limited to specific subgroup
Luspatercept + lenalidomide	Sekeres et al. (Phase Ib/II study, registered at ClinicalTrials.gov, NCT04539236)	Phase Ib results suggest efficacy	Investigating potential synergy in lower risk, non‐del(5q) MDS patients	Early‐stage study; needs further validation

*Note*: This table summarizes studies evaluating the efficacy of combination therapies involving ESAs and G‐CSF in patients with LR‐MDS. Combination therapy: ESA combined with G‐CSF to enhance erythroid response. Study details and patient population: key study references, sample size and patient characteristics (e.g. ESA‐naïve or ESA‐failure). Efficacy (response rate): reported erythroid response rates from each study. Key findings and benefits: main study conclusions, including potential benefits of combination therapy. Limitations: constraints such as small sample size, retrospective design or heterogeneous patient populations.

Abbreviations: EPO, erythropoietin; ESAs, erythropoiesis‐stimulating agents; G‐CSF, granulocyte colony‐stimulating factor; HI, haematological improvement; LR‐MDS, low‐risk myelodysplastic syndromes; MDS, myelodysplastic syndromes; RS, ring sideroblasts.

## APPROACH TO NTD ANAEMIA IN LR‐MDS PATIENTS (FIGURE 1A)

**FIGURE 1:**
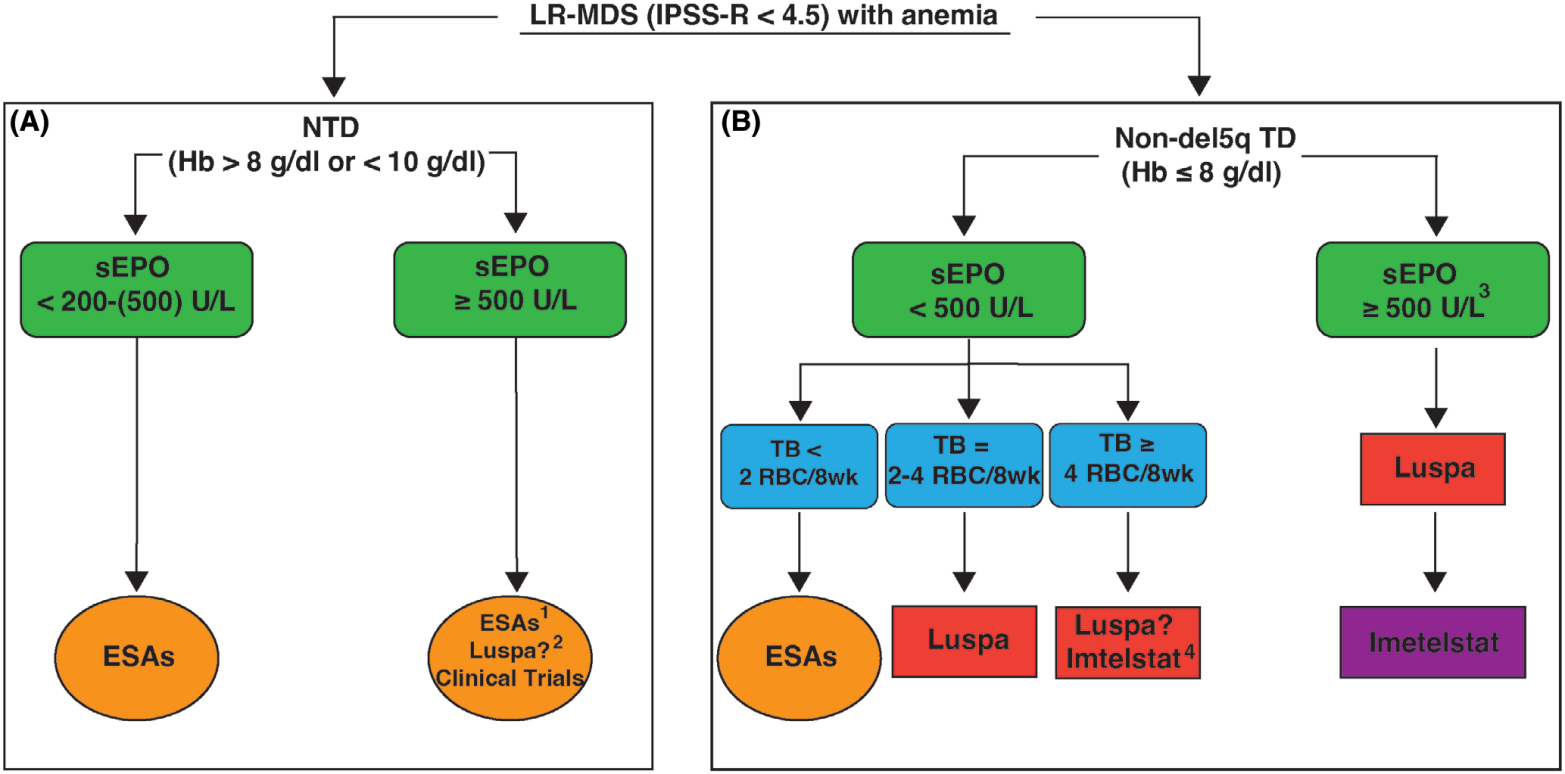
Therapeutic algorithm for anaemia in non‐del(5q) LR‐MDS: (A) approach to non‐transfusion‐dependent anaemia; (B) approach to transfusion‐dependent anaemia. ^1^Treatment with ESAs can be attempted in TI LR‐MDS patients with sEPO ≥500; however, response rates are limited, and the goal is to delay the occurrence of transfusion dependence. ^2^Data from the ongoing ELEMENT trial, which directly compares luspatercept with epoetin alfa in NTD LR‐MDS patients, is still awaited. ^3^Luspatercept is effective particularly in individuals with MDS‐RS or *SF3B1* mutation. ESAs, erythropoiesis‐stimulating agents; Hb, haemoglobin; IPSS‐R, Revised International Prognostic Scoring System; LR‐MDS, low‐risk myelodysplastic syndromes; Luspa, luspatercept; NTD, non‐transfusion dependent; RBC, red blood cell unit; sEpo, serum erythropoietin; TB, transfusion burden; TD, transfusion dependent.

Non‐transfusion‐dependent anaemia of patients with all subtypes of LR‐MDS should be considered for treatment only when symptomatic, which means that it could be difficult to point to a specific threshold of Hb, given the possible impact on the occurrence of symptoms of comorbidities, especially cardiac. Nevertheless, the approval of ESA indicates 10 g/dL as the level below which the drug can be prescribed. The tolerance to anaemia is extremely variable, and in some cases, patients themselves do not demand treatment.

In Europe, ESAs represent the first‐line treatment option in NTD LR‐MDS, due to their proven activity, relatively low cost and the fact that they can be self‐administered at home, rendering this therapy highly practical. As mentioned above, ESA treatment should be incepted at right doses of 30–40 000 U/weekly, rather early in the clinical history of disease, as soon as the anaemia gives symptoms, to obtain optimal efficacy and after having verified eligibility of the single patient (sEPO levels <200 IU/L, tentatively 500 U/L), karyotype, bone marrow blasts, IPSS‐R risk. Therapy should be tapered to the minimal dose and interval, in order to maintain Hb levels in a range of maximal 11–12 g/dL. Especially during the first couple of months of therapy, Hb levels should be monitored strictly to avoid steep increase in values. On the opposite, in some cases, response in terms of Hb increase may be obtained late, even after 12 weeks of ESAs. Interruption of treatment provokes recurrence of anaemia.

For LR‐MDS who are not eligible for ESA treatment, at present there are no approved alternatives, although androgens, despite risk of virilization and liver enzyme elevation, have shown some activity in mild–moderate anaemia.^80^ For LR‐MDS with del5q, low dose‐lenalidomide has been shown to have efficacy in delaying transfusion dependence.^81^ As mentioned above, an ongoing study is currently evaluating the activity of luspatercept versus ESAs in ESA‐naïve, NTD LR‐MDS and the outcome of this investigation could be relevant especially in patients with elevated sEPO and higher IPSS‐M.^52^

## APPROACH TO TRANSFUSION‐DEPENDENT ANAEMIA IN LR‐MDS PATIENTS (FIGURE 1B)

### ESAs refractory/resistant TD LR‐MDS non‐del(5q) patients

Treatment choice in TD hinges on two factors: the still impact of sEPO levels and the burden of transfusions.

- sEPO (<200–500 IU/L): In this subgroup, ESAs can still be considered in first‐line active treatment, due to their demonstrated ability to reduce transfusions in a proportion of patients whose transfusion need is recent and not heavy (<2 RBC U/8 weeks). The cost‐effectiveness and accessibility of ESAs further reinforce their possible positive role in this setting.
- sEPO (≥500 IU/L): In TD patients with elevated sEPO levels, as known since the Nordic score definition, ESAs are not active, while luspatercept is effective, particularly in individuals with MDS‐RS or *SF3B1* mutation. Clinical studies^60^, ^61^ have demonstrated that luspatercept can significantly reduce transfusion dependence in these patient populations, making it an essential option.

### ESA naïve TD LR‐MDS patients

According to evidence, luspatercept is more effective in inducing TI ≥12 weeks when compared to ESAs.^64^ Nevertheless, taking into account mode of administration and costs, ESAs could still be proposed to patients with a transfusion need <4 RBC units per 8 weeks (defined as low transfusion burden [LTB]) where achievement of TI was 63.1% versus 25.7% of those with a transfusion need ≥4 RBC units per 8 weeks (defined as HTB). In the EPOANE study,^13^ results were consistent, and erythroid response was obtained in 25% of TD LR‐MDS patients versus 66.7% of NTD patients. Indeed, in patients with HTB, luspatercept induces TI in 50% of cases versus 78% obtained in patients with LTB. Most relevantly, duration of response in LTB cases is 126 weeks versus 91 weeks for luspatercept treatment versus epoetin alfa respectively.^82^

## DISCUSSION

There is a lively discussion on how to schedule and sequence ESAs and luspatercept in the treatment of anaemic LR‐MDS patients both in terms of efficacy and costs. Without any doubt, present evidence confirms the choice to use ESAs in NTD LR‐MDS patients with symptomatic anaemia at doses ranging from 30 000 to 60 000 U/week. However, challenges arise when patients are ineligible for ESAs or become refractory, or have lost response but are not yet TD. In this setting, we do not have at present alternative therapies. The ongoing study comparing ESAs to luspatercept in NTD LR‐MDS will in part provide answers for specific subsets of patients, provided the study is designed and powered to verify the activity in specific subsets of LR MDS patients.

The availability of luspatercept, approved for TD LR‐MDS‐RS and more recently approved also for all subtypes of TD MDS,^66^, ^67^ has provided an important therapeutic tool. Indeed, in the setting of transfusion‐dependent patients, where ESAs are very well known to be scarcely effective, the recommendation is to use luspatercept. The dose of luspatercept is to be adjusted upon verification of response and may be escalated to 1.75 mg/kg/3 weeks. At this moment, the utility of the maximal dose of luspatercept implemented since the beginning of therapy is under evaluation in two ongoing clinical studies, the MAXILUS trial (registered at ClinicalTrials.gov, NCT06045689) and the LUSPLUS trial (registered at ClinicalTrials.gov, NCT05181592).

Moreover, while investigational studies^60^, ^69^, ^70^, ^71^ have demonstrated the effectiveness of luspatercept as therapy in TD LR‐MDS patients after ESAs, no data have been currently produced on the efficacy of ESAs as second‐line treatment after luspatercept failure, which leaves clinicians without guidance for managing these patients, whose number is increasing over time. One has to remind that MDS‐RS cases, although achieving TI in a high percentage of cases, may show a transient response, although a long one, and will need further therapies, given their prolonged OS.^83^ Imetelstat can provide this salvage opportunity, as recently observed.^84^

The use of luspatercept in combination with other agents and also in MDS‐del(5q) is an avenue to be explored. The attention of some researchers is at present focused on possible treatment with luspatercept of the small number of lenalidomide refractory or relapsed patients (registered at ClinicalTrials.gov, NCT05924100), but data are not yet available.

To refine possible treatment algorithms (Figure 1) for LR‐MDS patients with anaemia, who constitute the majority of MDS patients walking in our clinics, we have to ensure that both therapies are chosen optimally, based on patient clinical and biological characteristics. To achieve this goal, not yet completely feasible, we need to conduct further studies, and especially academically driven investigations, determining the variables associated with response for each drug and focusing on the optimal sequencing or combination strategies of the two agents. These predictors of response should be easily measurable in routine practice with widely accessible texts. Other molecules are at the horizon for TD LR‐MDS patients. Imetelstat, a specific telomerase inhibitor, has just been approved by EMA in this setting.^85^ We have to get ready to implement our standards of treatment to offer the best quality of therapy for our patients.

## CONFLICT OF INTEREST STATEMENT

VS has participated in advisory boards for Abbvie, Ascentage, BMS, Geron, Jazz, Curis, Keros, Novartis, Servier, Syros and has received travel grants from Abbvie, Jazz, Janssen. AC declares no competing financial interests related to this work.
